# A proprietary black cumin oil extract (*Nigella sativa*) (BlaQmax^®^) modulates stress-sleep-immunity axis safely: Randomized double-blind placebo-controlled study

**DOI:** 10.3389/fnut.2023.1152680

**Published:** 2023-04-17

**Authors:** Muttanahally Eraiah Mohan, Jestin V. Thomas, Mohind C. Mohan, Syam Das S, Prathibha Prabhakaran, Baby Chakrapani Pulikkaparambil Sasidharan

**Affiliations:** ^1^Department of General Medicine, BGS Global Institute of Medical Sciences, Bengaluru, Karnataka, India; ^2^Leads Clinical Research and Bio Services Private Limited, Bengaluru, Karnataka, India; ^3^Centre for Neuroscience, Cochin University of Science and Technology, Kochi, Kerala, India; ^4^R&D Centre, Akay Natural Ingredients, Kochi, Kerala, India; ^5^Department of Biotechnology, Cochin University of Science and Technology, Kochi, Kerala, India

**Keywords:** black cumin, Pittsburgh Sleep Quality Index, Perceived Stress Scale, stress, non-refreshing sleep, sleep quality, immunity

## Abstract

**Objective:**

Stress, sleep, and immunity are important interdependent factors that play critical roles in the maintenance of health. It has been established that stress can affect sleep, and the quality and duration of sleep significantly impact immunity. However, single drugs capable of targeting these factors are limited because of their multi-targeting mechanisms. The present study investigated the influence of a proprietary thymoquinone-rich black cumin oil extract (BCO-5) in modulating stress, sleep, and immunity.

**Methods:**

A randomized double-blinded placebo-controlled study was carried out on healthy volunteers with self-reported non-refreshing sleep issues (*n* = 72), followed by supplementation with BCO-5/placebo at 200  mg/day for 90  days. Validated questionnaires, PSQI and PSS, were employed for monitoring sleep and stress respectively, along with the measurement of cortisol and melatonin levels. Immunity markers were analyzed at the end of the study.

**Results:**

In the BCO-5 group, 70% of the participants reported satisfaction with their sleep pattern on day 7 and 79% on day 14. Additionally, both inter- and intra- group analyses of the total PSQI scores and component scores (sleep latency, duration, efficiency, quality, and daytime dysfunction) on days 45 and 90 showed the effectiveness of BCO-5 in the improvement of sleep (*p* < 0.05). PSS-14 analysis revealed a significant reduction in stress, upon both intra (*p* < 0.001) and inter-group (*p* < 0.001) comparisons. The observed reduction in stress among the BCO-5 group, with respect to the placebo, was significant with an effect size of 1.19 by the end of the study (*p* < 0.001). A significant correlation was also observed between improved sleep and reduced stress as evident from PSQI and PSS. Furthermore, there was a significant modulation in melatonin, cortisol, and orexin levels. Hematological/immunological parameters further revealed the immunomodulatory effects of BCO-5.

**Conclusion:**

BCO-5 significantly modulated the stress-sleep-immunity axis with no side effects and restored restful sleep.

## 1. Introduction

Stress, insomnia, and immunity are the three major interconnected factors, that play key roles in the maintenance of health. It has been established that stress can negatively affect the quality and duration of sleep, which in turn impacts immunity (1). Stress, a mental condition generated by either extrinsic or intrinsic factors, may lead to various psychological, biological, and/or social issues. It has been identified as a major factor associated with sleep disorders. Since sleep is essential for energy, cell tissue repair, metabolic regulation, thermoregulation, cognition, motor actions, and immune functions (1, 2), it has been proposed that the ‘stress-sleep-immunity’ axis is critical for health (2).

Sleep and stress share multiple pathways and affect the central nervous system and circadian rhythm, leading to dysfunctions in metabolism, brain function and immunity (3). The hypothalamus-pituitary–adrenal axis (HPA) is the central system responsible for the neuroendocrine adaptation of the stress response. Orexins or hypocretins (Orexin A and Orexin B) are excitatory neuropeptides produced in the hypothalamus in response to stress stimuli (4). It modulates the activity of the HPA axis and autonomic nervous system to regulate the sleep–wake cycle, cognitive functioning, stress processing, and the metabolic and inflammatory responses (4). Orexins are also responsible for the release of cortisol, a major hormone released in response to stress (4).

Sleep and immune functions are interconnected. The sleep–wake cycle is one of the most important manifestations of the circadian rhythm, and its changes affect physical and mental activities, major organ functions, temperature regulation and immunomodulatory effects such as leukocytes and cytokine production and proliferation (1). It has been reported that one to three nights of sleep deprivation can lead to a significant elevation of inflammatory cytokines and can significantly reduce leukocyte, lymphocyte, and neutrophil counts (5).

Despite the importance of stress, sleep, and immunity, drugs/supplements capable of safely modulating the stress-sleep-immunity axis are limited because they act *via* multi-targeted mechanisms and side effects, including dependency, are common. *Nigella sativa*, commonly known as black cumin or black seed, is a culinary spice and an age-old medicinal herb with a wide range of health-beneficial pharmacological effects to attenuate oxidative stress, inflammation, immunity, energy metabolism, and cell survival (6, 7). Black cumin oil composed of both volatile and fixed oil fractions was identified as the major bioactive component of black cumin, in which thymoquinone (TQ) is considered the most active molecule (8). Black cumin has been reported to have antioxidant, anti-inflammatory, and neuroprotective effects as a function of its TQ content (6, 9–11). Recently, it was also reported that a novel formulation of black cumin oil extract containing TQ and carvacrol in a 10:1 (w/w) ratio (BCO-5) significantly alleviated stress and improved sleep quality in human volunteers when supplemented at 200 mg/day for 28 days (12). Jestin et al. conducted a 90-days safety assessment of BCO-5 among healthy human volunteers and established its safety for human consumption (13).

Based on previous studies, we hypothesized that BCO-5 would modulate the stress-sleep-immunity axis by reducing stress, improving sleep quality, and hence, the immunity. Thus, the present randomized, double-blinded, placebo-controlled study investigated the efficacy of BCO-5 in healthy subjects (25–65 years old) with significant stress and non-restorative sleep. Validated questionnaires were employed to analyze sleep quality [Pittsburgh Sleep Quality Index (PSQI)] and stress [Perceived Stress Scale (PSS)], along with changes in sleep and immune biomarkers.

## 2. Materials and methods

The proprietary formulation of black cumin oil used in this study (BCO-5; Patented and Registered as BlaQmax^®^) was manufactured by Akay Natural Ingredients, Cochin, India, following good manufacturing practices (Batch no: BCOQ 32/21 dated 12/04/2021). The dried black cumin seeds used for the manufacture of BCO-5 were identified and authenticated by a botanist, and the specimens were deposited at the Herbarium of Akay Natural Ingredients, Cochin, India (Voucher no: AK-NS-018). High-performance thin-layer liquid chromatography (HPTLC) (CAMAG HPTLC system, Switzerland) was employed to identify black cumin, as previously reported (13). The thymoquinone content was determined by high-performance liquid chromatography (HPLC) (Shimadzu Analytical India Private Limited, Mumbai, India) analysis, as reported previously (13). Analytical standard for TQ (CAS No: 490–91-5) was obtained from Sigma-Aldrich (Bangalore, India).

### 2.1. Subjects and design

**Figure 1:**
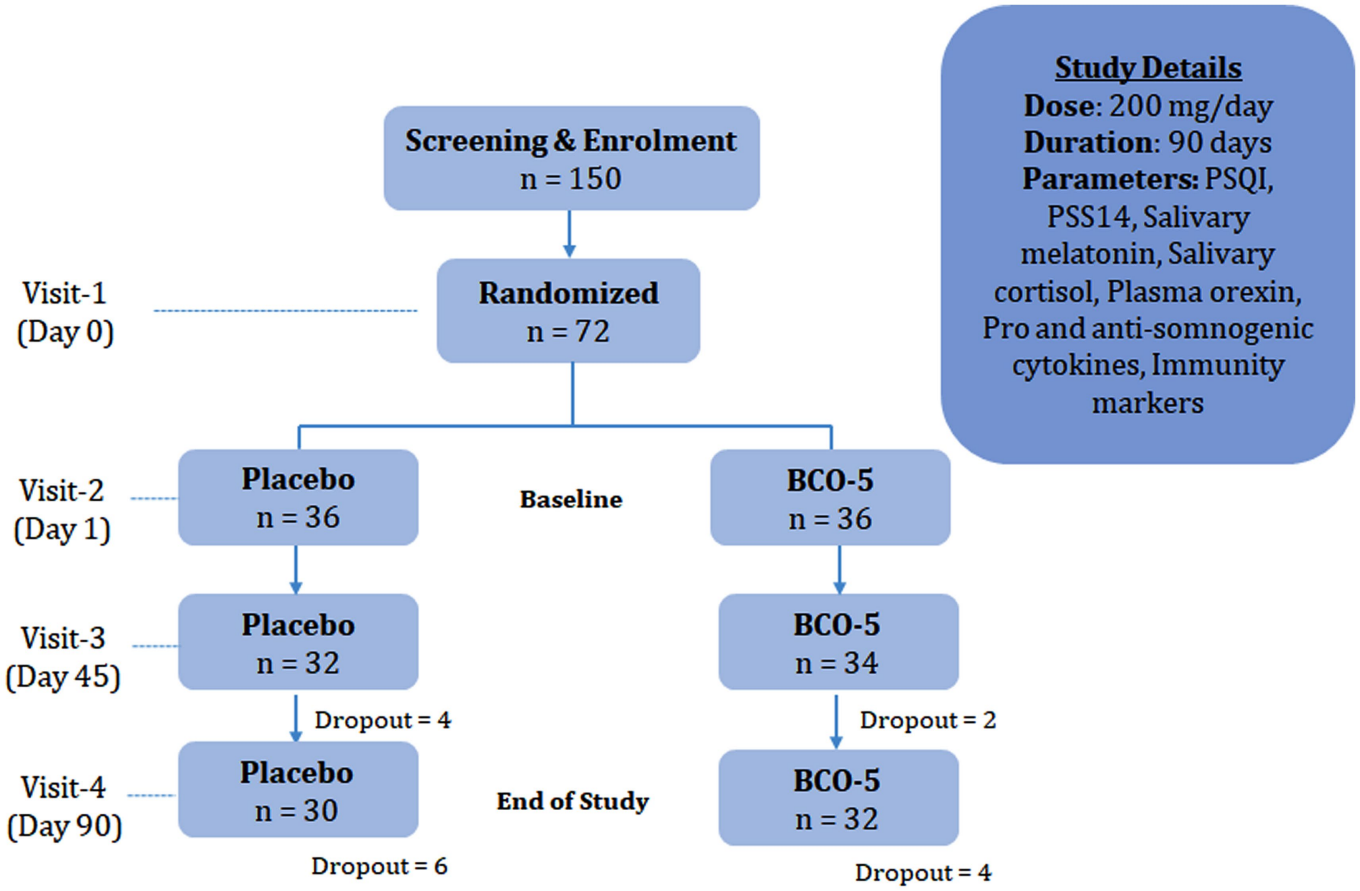
Cohort diagram depicting study design.

In this randomized, double-blinded, placebo-controlled clinical trial, 150 volunteers (aged 25 to 65 years; healthy subjects) who were reported to experience significant stress and self-reported sleep issues such as non-restorative sleep, waking up at night multiple times, or having difficulty in a sound sleep for the past 4 weeks were selected. Volunteers were identified from the database of the contract research organization and the hospital where the study was conducted. After the initial description of the study, 96 participants were willing to participate and were further screened according to the inclusion and exclusion criteria (Table 1). Twenty-four subjects were eliminated after screening, and 72 volunteers were randomized into BCO-5 and placebo groups (*n* = 36/group). The study was conducted at the BGS Global Institute of Medical Sciences, Bangalore, India, under the guidance of a registered medical practitioner, according to the guidelines of the Clinical Trial Registry of India (CTRI/2021/05/033780 dated 25/05/2021). The protocol was reviewed and approved by the institutional ethics committee. Written informed consent was obtained from all subjects prior to the study. A cohort diagram representing the study design is shown in Figure 1.

**Table 1:** Inclusion and exclusion criteria.

**Inclusion criteria** Healthy participants aged 25–65 years (both inclusive) Participants with PSQI scores ≥5 Participants with PSS score between 14 and 26 Participants with body weight ≥ 50 kg Approved birth control measures should be perceived by female participants of childbearing age and should have negative urine pregnancy test at the screening Participants should refrain from smoking, caffeinated beverages, and alcohol consumption Signed and informed consent should be provided by the participant and the participant should understand the study protocol
**Exclusion criteria** Participants requiring medical treatment and suffering from health conditions like hypertension, diabetes, chronic renal failure, heart, thyroid and liver disease Participants with hepatic or renal impairment (Alanine transaminase/Aspartate transaminase levels >3 upper limit of normal) (serum creatinine ≥2.0 mg/dl) Subjects with history of conditions such as endocrine abnormalities including thyroid disease, psychiatric illness, drug abuse, smoking, addiction to alcohol, psychiatric illness Participants who have went cardiovascular surgery or any other major surgery Immuno-compromised state participants and those with immunodeficiency diseases like, HIV or Hepatitis B Participants allergic to the composition of investigational product Pregnant and lactating women Participants with significant illness history or any medical derangements that can interfere with subject treatment, assessment, or compliance with the protocol Participants currently participating or have participated in any other clinical trial, 1 month prior to start of this study Any additional condition(s) that in the Investigators opinion would warrant exclusion from the study or prevent the subject from completing the study.

The participants were requested to visit the study site on four different occasions; Visit I (day 0), Visit II (day 1), Visit III (day 45) and Visit IV (day 90/ end of study). During visit I, screening was performed against the inclusion/exclusion criteria, which included a structured medical interview and diagnosis as well as demographic and anthropometric measurements. The primary selection criterion was a PSQI score greater than 5. During visit II (day 1), the participants were asked to report at the study site with 10 h of fasting and were randomized, based on a computer-generated randomization code, into two groups to receive either a placebo or intervention. Blood (10 mL) was withdrawn for routine laboratory clinical parameters (biochemical and hematological) and markers of immunity. The baseline PSQI and PSS-14 scores were also recorded. Similar visits for blood and data collection were requested on day 45 (Visit III) and at the end of the study period, day 90 (Visit IV). Telephonic interviews were also performed on days 7 and 14 to inquire about adverse events, side effects, tolerance, and efficacy.

### 2.2. Sample size and randomization

Sample size calculation was performed using the G Power Statistical Software (3.1.9.7 Version, Franz Faul, University of Kiel, Kiel, Germany) (14). It was estimated that a total of 35 participants per group would be required, with an anticipated non-compliance or dropout rate of 20%, yielding 80% power, and 5% significance level. Participants were randomly allocated by permuted-block randomization (block size = 4) using a computer-generated allocation table.^1^

### 2.3. Intervention and dosage

Airtight high density polyethylene containers comprising 95 soft gel capsules (200 ± 10 mg extract/capsule) with either BCO-5 (intervention) or placebo were sequentially coded and provided to the participants. They were requested to consume one capsule daily after dinner, 20–30 min prior to bedtime. The total study duration was 90 ± 2 days. At the end of the study period (Visit IV), the remaining capsules were recorded. The effectiveness of participant blinding was evaluated by asking participants to predict the allocation (placebo/intervention).

### 2.4. Pittsburgh Sleep Quality Index

The PSQI is a 19- item self-reported questionnaire designed to determine the overall sleep quality and disturbances over a period of one-month tenure. Four-point Likert scale is used to rate the severity/frequency of the problems in such a way that ‘0’ = not during the past month, ‘1’ = less than once in a week, ‘2’ = Once or twice a week, ‘3’ = Three or more times a week. Each component yields a score ranging from 0 to 3, and the component scores are summed to yield a global PSQI score (from 0 to 21). A higher score indicates lower sleep quality (15). A score greater than 5indicates poor sleep quality or insomnia (16).

### 2.5. Perceived Stress Scale- 14 (PSS-14)

The PSS questionnaire was developed by Cohen et al. in 1983 to evaluate the perception of stressful conditions in a person’s life based on their last one-month experience. It is the degree to which situations in life are appraised using a 14-item scale. A 5-point Likert scale, ranging from 0 (never) to 4 (very often), was used to evaluate the stress experienced. The PSS scores ranges from 0 to 56; with higher scores indicating higher perceived stress (17). Scores ranging to 0–18 indicate low stress; to 19–37 implies moderate, and to 38–56 as high stress.

### 2.6. Sleep and stress biomarkers

#### 2.6.1. Estimation of salivary cortisol and melatonin

Cortisol and melatonin levels from the salivary samples were estimated using ELISA Kit methods of Neogen Corporation, KY, United States (Catalog No: 402710) and IBL-International, Germany (Catalog No: RE54041), respectively. Micro ELISA 96 well plates were used for the analysis. Absorbance was measured at 450 ± 2 nm using a Varioskan^™^ LUX multimode microplate reader (Thermo Scientific^™^, Waltham, MA, United States).

#### 2.6.2. Orexin A assay

Plasma Orexin A levels were estimated using an ELISA kit method (Catalog No: E-EL-H1015) (Elabscience, Biotechnology Co., Limited Bethesda, United States). Measurements were conducted in Micro ELISA 96 well plates using a Varioskan^™^ LUX multimode microplate reader (Thermo Scientific^™^, Waltham, MA, United States) at 450 ± 2 nm.

### 2.7. Immunity biomarkers

#### 2.7.1. Analysis of immunoglobulins

Serum immunoglobulin concentrations of IgG (Catalog no: E-EL-H0169) and IgM (Catalog no: E-EL-H1814) were analyzed using the ELISA kit method following the manufacturer’s instructions at 450 ± 2 nm, using a Varioskan^™^ LUX multimode microplate reader (Thermo Scientific^™^, Waltham, MA, United States).

#### 2.7.2. CD4+, CD8+ absolute count

Whole blood samples were collected in EDTA tubes, and 50 μL of the sample was added to a test tube containing a pre-dispensed, stabilized monoclonal antibody. The antibody mixture containing antibodies against CD4 and CD8 was provided by Becton Dickinson (San Diego, CA, United States) and conjugated with allophycocyanin (APC) and fluorescein isothiocyanate (FITC). The blood sample was diluted (1:10) using phosphate buffer after 15 min of incubation. The samples were analyzed using flow cytometry (FACS Calibur, Becton Dickinson, CA, United States). The sample analysis was performed in comparison with a specific fluorescence signal attributed to the presence of CD4 and CD8 antigens at the cell surface, with a side scatter signal for discriminating cells based on their shape and structure. The method of determination of absolute count was determined as described by Arneth (18).

#### 2.7.3. Differential count

Whole blood sample were collected from the antecubital vein. The total and differential WBC counts were analyzed using automated cell cytometry (Quest Diagnostics, Inc., Madison, New Jersey, United States). Another 3 ml of whole blood was collected in EDTA vials and kept at room temperature until analysis within 24 h of sampling.

#### 2.7.4. Analysis of cytokines

Serum concentrations of IL-1β (catalog no: E-EL-H0149), IL-2 (catalog no: E-EL-H0099), and IL-10 (catalog no: E-EL-H6154) were analyzed using ELISA kits, according to the manufacturer’s instructions. Kits were purchased from Elabscience Biotechnology Co., Limited. Bethesda, United States. Absorbance was read at 450 ± 2 nm using a Varioskan^™^ LUX multimode microplate reader (Thermo Scientific^™^, Waltham, MA, United States).

### 2.8. Statistical analysis

Statistical analyses were performed using SPSS version 27.0. A 2 × 2 repeated measures ANOVA was employed to analyze statistical significance (treatment vs. time). Bonferroni test was used to adjust for multiple comparisons. The significance of the difference is represented as a ‘*p*’ value. *p* < 0.05 was considered statistically significant. The reported values are arithmetic means with standard deviations (SD) or standard errors of the mean (SEM) as indicated. Pearson’s correlation test was used to evaluate the significance of correlation between sleep quality (PSQI) and stress (PSS-14).

## 3. Results

### 3.1. Materials, subjects, and study design

HPTLC analysis confirmed that raw material used for preparing BCO-5 was *Nigella sativa* seed. It was in an oil form with 5.12% TQ content and 0.54% carvacrol in such a way that the TQ to carvacrol content ratio was 1:10. BCO-5 was food-grade and free from synthetic emulsifiers and food contaminants such as pesticides, heavy metals, mycotoxins, polyaromatic hydrocarbons, ethylene oxide, and microbial pathogens, as evident from their certificate of analysis.

The baseline anthropometric, haemodynamic, and other vital characteristics of the placebo and BCO-5 treated groups are provided in Supplementary Table S1. The average age and BMI of the participants in the placebo group were 25.76 ± 15.51 and 23.75 ± 0.79, respectively, while those of the BCO-5 group were 24.5 ± 16.62 and 24.29 ± 0.72, respectively. At the end of the study period, there were no significant differences between these factors.

No major side effects or adverse events were reported during the telephonic interviews on days 7 and 14. Three participants from the BCO-5 group and one from the placebo group reported bloating and borborygmus with a taste of oil in the mouth at different instances. However, all of them continued since they were satisfied with the improvement in their sleep quality. By day 14, no adverse events had been reported, and none of the participants showed signs of sleepiness or daytime drowsiness with fatigue. During the study period, 92% of participants in the BCO-5 group and 78% in the placebo group were found to be using supplements, although the consistency of use over 90 days could not be ascertained.

### 3.2. Influence of BCO-5 on sleep

**Figure 2:**
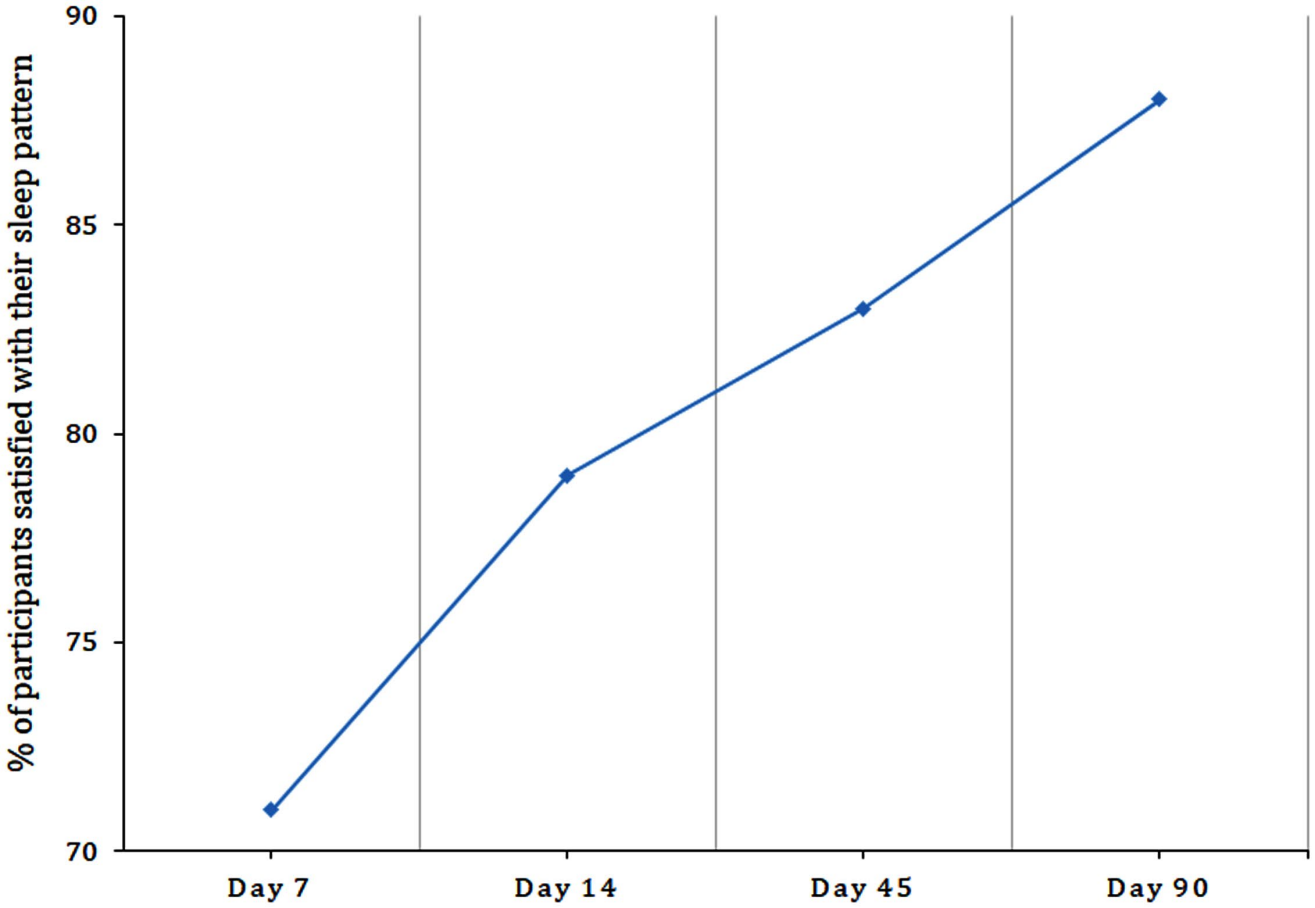
Graphical representation of the percentage of participants satisfied with their sleep pattern upon supplementation with BCO-5 as observed from sleep diary.

Approximately 62% of the participants reported the beneficial effect of a single dosage. Upon telephonic interview on day 7, about 70% of participants reported satisfaction, which increased to 79% by day 14 (Figure 2). Reduction in sleep disturbances and better sleep were the two main types of feedback that were received.

#### 3.2.1. PSQI on day 45

**Figure 3:**
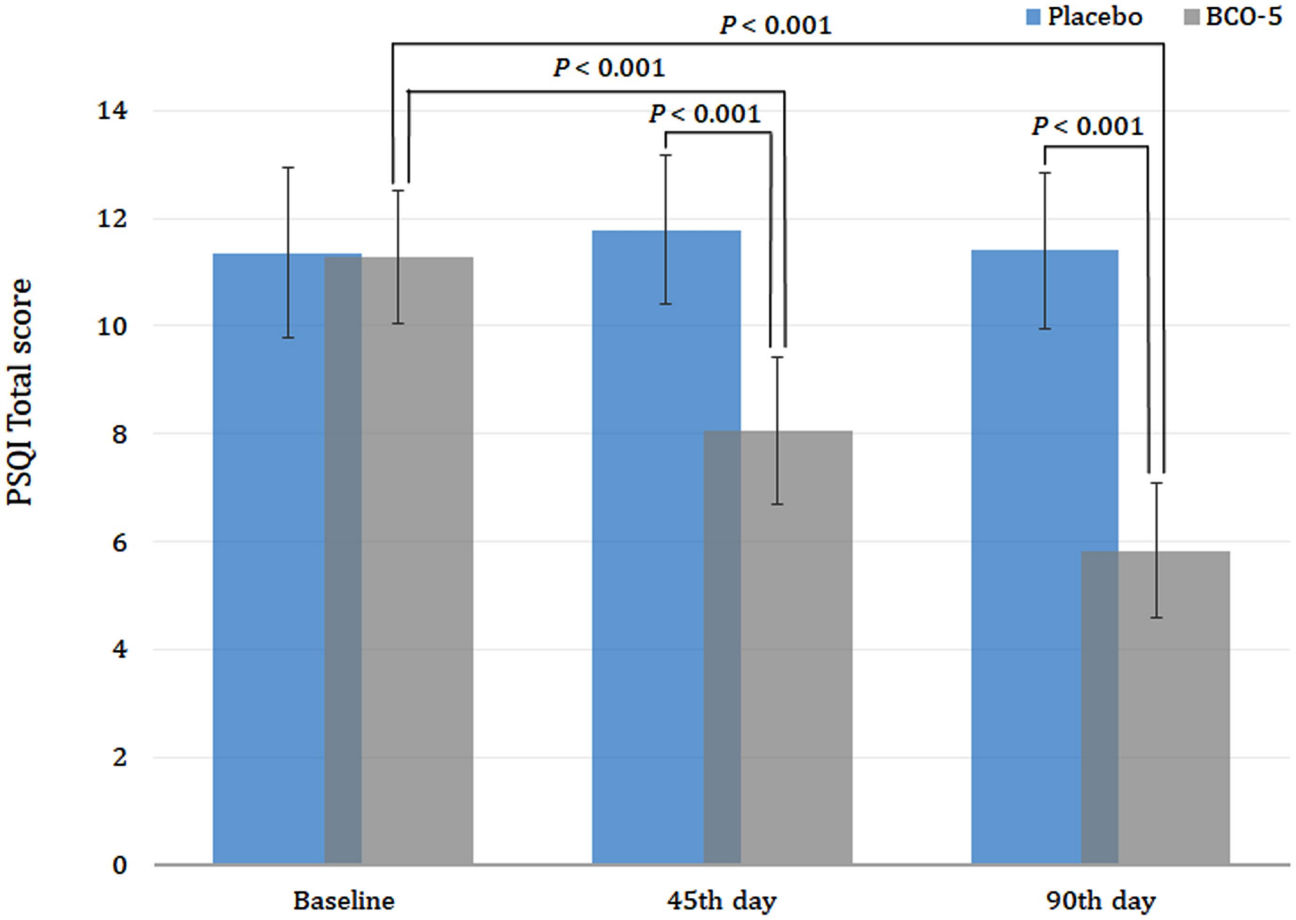
Effect of BCO-5 on PSQI total scores on 45th day 90th day compared to Placebo. The values are expressed as mean ± SD.

**Figure 4:**
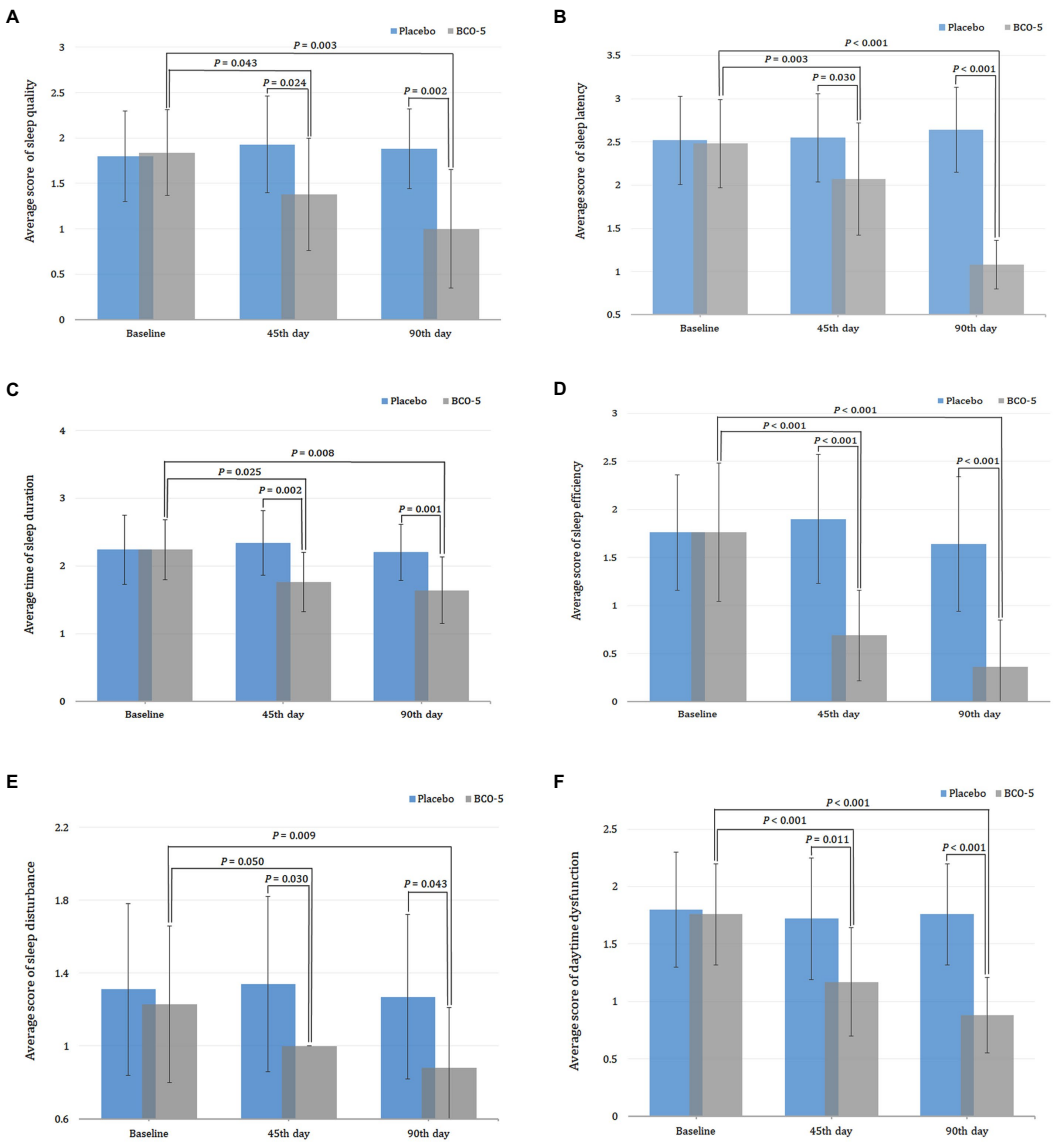
Relative changes in PSQI component scores. **(A)** Sleep quality. **(B)** Sleep latency. **(C)** Sleep duration. **(D)** Sleep efficiency. **(E)** Sleep disturbance. **(F)** Daytime dysfunction, when supplemented with BCO-5 on 45th day and 90th day compared to placebo. The values in **(B,C,E,F)** are provided as mean ± SD.

Intra-group comparison (baseline versus day 45) of the PSQI total score revealed a significant reduction (*p* < 0.001) in the BCO-5 group and a non-significant reduction in the placebo (Figure 3). Detailed analysis further revealed relative changes in various component scores corresponding to sleep quality (*p* = 0.043), sleep latency (*p* = 0.003), sleep duration (*p* = 0.025), overall sleep efficiency (*p* < 0.001), sleep disturbance (*p* = 0.050) and daytime dysfunction (*p* < 0.001) among BCO-5 participants. However, changes in sleep parameters were not significant in the placebo group (Figures 4A–F).

Inter-group comparison (Placebo versus BCO-5) of the PSQI component scores at baseline showed no significant difference (*p* > 0.05) between the BCO-5 and placebo. However, supplementation with BCO-5 resulted in a significant reduction in the PSQI total score (large difference effect size of 0.68; 95% CI: 7.55–8.59; *p* < 0.001). The overall effect size observed for various parameters were: sleep quality- 0.31 (95% CI: 1.14–1.61; *p* = 0.024), sleep latency- 0.31 (95% CI: 1.82–2.31; *p* = 0.030), sleep duration- 0.24 (95% CI: 1.59–1.92; *p* = 0.002), sleep efficiency- 0.52 (95% CI: 0.51–0.86; *p* < 0.001), sleep disturbance- 0.19 (95% CI: 1.00–1.00; *p* = 0.030), and daytime dysfunction- 0.29 (95% CI: 0.99–1.35; *p* =0.011) compared to placebo (Figures 4A–F; Table 2). All component scores exhibited a moderate to large difference effect on the 45th day when compared to the placebo.

**Table 2:** Effect size and *p*-values of PSQI parameter (BCO-5 vs. Placebo) on day 45 and 90.

PSQI parameter	Time	Effect size	*P*-value
Sleep quality	45th day	0.31	0.024
	90th day	0.57	0.002
Sleep latency	45th day	0.31	0.030
	90th day	0.82	<0.001
Sleep duration	45th day	0.24	0.002
	90th day	0.33	0.001
Sleep efficiency	45th day	0.52	<0.001
	90th day	0.47	<0.001
Sleep disturbance	45th day	0.19	0.030
	90th day	0.20	0.043
Daytime dysfunction	45th day	0.29	0.011
	90th day	0.56	<0.001
Total PSQI	45th day	0.68	<0.001
	90th day	0.86	<0.001

A significance level *p* < 0.05 is considered as statistically different.

#### 3.2.2. PSQI on day 90

By the end of the study period (day 90), BCO-5 supplementation revealed a continuous improvement in sleep parameters with a significant reduction in the PSQI total score and component scores on both intra and inter-group comparison. The total PSQI score of BCO-5 was significantly lower than that at baseline (*p* < 0.001) (Figure 3). The relative improvement in component scores for BCO-5 in comparison with baseline was sleep quality (*p* = 0.003), sleep latency (*p* < 0.001), sleep duration (*p* = 0.008), sleep efficiency (*p* < 0.001), sleep disturbance (*p* = 0.009) and daytime dysfunction (*p* < 0.001) (Figures 4A–F). However, the relative changes in the placebo group were not significant (*p* > 0.05).

Overall effect size observed for various component scores were: sleep quality– 0.57 (95% CI: 0.73–1.26; *p* = 0.002), latency– 0.82 (95% CI: 0.96–1.19; *p* < 0.001), duration– 0.33 (95% CI: 1.43–1.84; *p* = 0.001), efficiency– 0.47 (95% CI: 0.15–0.56; *p* < 0.001), disturbance– 0.20 (95% CI: 0.75–1.01; *p* = 0.043), daytime dysfunction– 0.56 (95% CI: 0.74–1.01; *p* < 0.001) and total PSQI– 0.86 (95% CI: 5.32–6.35; *p* < 0.001) (Figures 4A–F and Table 2).

Intra- and inter-group comparisons of the PSQI data corresponding to days 45 and 90 was also compared using paired and independent t-tests and were found to be significant (Supplementary Table S2).

### 3.3. Influence of BCO-5 on stress

**Figure 5:**
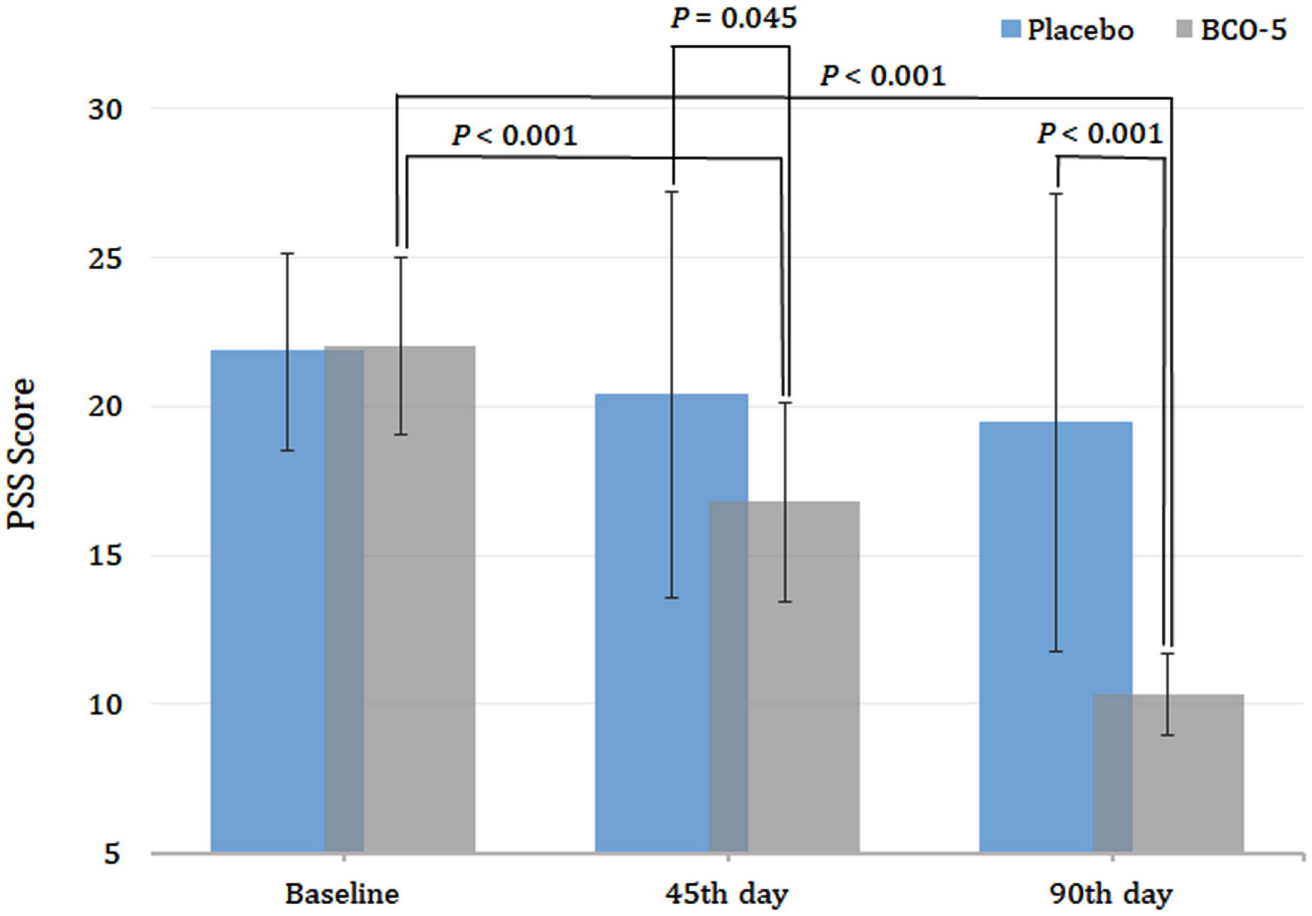
Relative changes in PSS-14 score upon supplementation with BCO-5 on day 45 and 90 compared to placebo. The values are expressed as mean ± SD.

Intra-group comparison of PSS-14 scores on the 45th day revealed a significant reduction (*p* < 0.001) in the BCO-5 group, whereas the placebo showed no significant change. Inter-group comparison also showed a significant reduction in stress in the BCO-5 group compared to the placebo. The effect size observed was 0.17 (95% CI: 15.51–18.06; *p* = 0.045) (Figure 5).

Both intra- and inter-group comparisons showed a significant effect of BCO-5 at the end of the study (day 90). The effect size observed was moderate effect of 0.43 (95% CI: 9.76–10.87; *p* < 0.001) (Figure 5). The data were also analyzed using paired and independent *t*-tests and were found to be significant (Supplementary Table S3).

### 3.4. Pearson’s correlation between sleep and stress

BCO-5 administration showed a negative correlation between the PSQI and PSS-14 measures, with a coefficient of −0.314. This correlation was significant at the end of the study (*p* < 0.05). In contrast, placebo administered group exhibited a positive correlation between PSQI and PSS-14 parameters at the end of the study (0.178; *p* = 0.357).

### 3.5. Effect of BCO-5 on melatonin level

**Figure 6:**
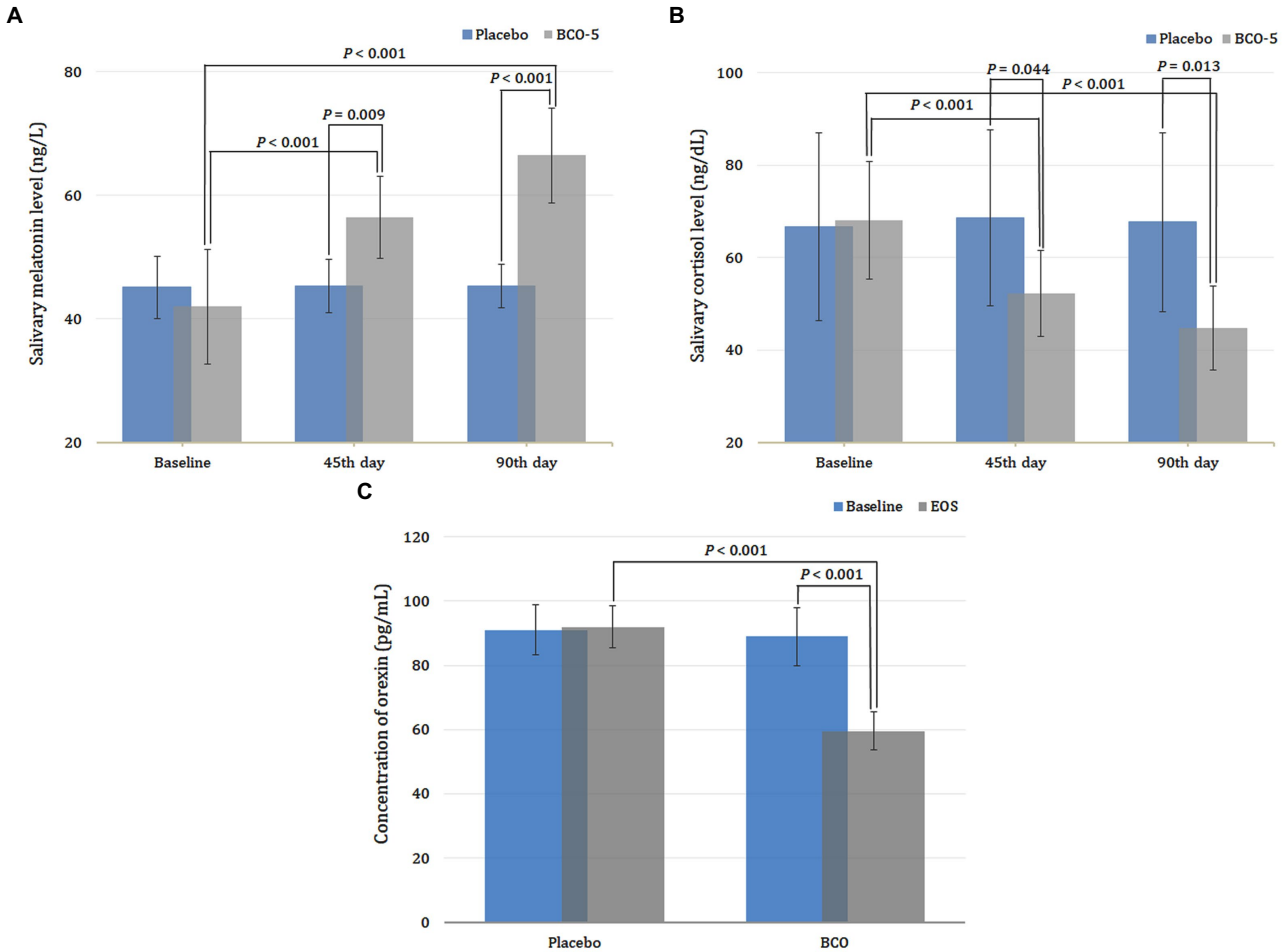
Changes in sleep biomarkers **(A)** melatonin and **(B)** cortisol and **(C)** orexin A on 45th day and 90th when supplemented with BCO-5 when compared to placebo. The values are expressed as mean ± SD.

Both intra and inter-group analyses of salivary melatonin on day 45 showed no significant increase in placebo (*p* > 0.05), whereas a significant increase in the BCO-5 group was observed in both intra- (*p* < 0.001) and inter-group analyses (*p* = 0.009). The overall effect size was 0.84 (95% CI: 53.96–58.93) (Figure 6A). When extended to day 90, the placebo group exhibited no significant increase in melatonin levels while BCO-5 had a significant effect on both intra- (*p* < 0.001) and inter-group comparisons (*p* < 0.001). A large effect size [0.85 was observed at the end of the study (95% CI: 63.32–69.66) (Figure 6A)].

### 3.6. Effect of BCO-5 on cortisol level

The intra- and inter-group analysis of cortisol at the end of the 45th day exhibited a significant decrease (*p* < 0.001) for BCO-5, while the placebo showed no significant change (*p* > 0.05). The effect size observed upon analysis was 0.72 (95% CI: 48.73–55.70; *p* = 0.044).

At the end of the study (day 90), BCO-5 participants showed a significant decrease (*p* < 0.001), while the placebo exhibited no significance (*p* > 0.05) with respect to the baseline. Inter-group comparison at the end of the study also revealed a significant decrease in cortisol levels among BCO-5 participants compared to placebo. A large effect of 0.72 (95%CI: 40.99–48.51; *p* = 0.013) was observed (Figure 6B).

### 3.7. Effect of BCO-5 on orexin level

At the end of the study, the plasma concentration of orexin in the BCO-5 group, decreased significantly in upon both intra- and inter-group comparisons (*p* < 0.001), while the same for the placebo exhibited no significant change. The observed effect size had a large difference effect of 0.92 (95% CI: 57.07–61.99) (Figure 6C).

### 3.8. Effect of BCO-5 on immune markers

#### 3.8.1. Influence on immunoglobulins

Intra-group analysis of IgM and IgG at the end of the study period revealed a significant increase compared to baseline (*p* < 0.008 and *p* < 0.001, respectively), whereas the placebo showed no significant effect. Inter-group analysis of IgM and IgG levels also showed a significant increase compared to the placebo. A moderate effect size of 0.41 (95% CI: 191.06–214.24; *p* = 0.050) was noted for IgM, and a large effect size of 0.87 (95% CI: 1091.09–1125.94; *p* < 0.001) for IgG (Table 3).

**Table 3:** Changes in immunity markers in participants from baseline to end of study in placebo and BCO-5 treated group and the *p*-values observed upon inter-group and intra-group comparison.

Parameters	Group	Baseline	End of study	*P*-values	
				Inter	Intra
IgG (mg/dL)	Placebo	848.04 ± 56.63	850.72 ± 52.53	<0.001	<0.001
	BCO-5	818.12 ± 63.65	1108.52 ± 42.22		
IgM (mg/dL)	Placebo	173.50 ± 24.55	168.15 ± 36.08	0.050	0.008
	BCO-5	174.15 ± 45.03	202.65 ± 28.69		
CD4 + cells	Placebo	655.91 ± 81.51	639.27 ± 91.90	<0.001	0.015
	BCO-5	703.18 ± 102.93	792.05 ± 60.84		
CD8 + cells	Placebo	398.32 ± 55.27	413.45 ± 57.10	0.042	0.043
	BCO-5	446.05 ± 34.18	406.91 ± 42.87		
CD4/CD8 ratio	Placebo	1.63 ± 0.33	1.57 ± 0.29	0.032	<0.001
	BCO-5	1.60 ± 0.23	1.97 ± 0.26		

Serum levels of immunoglobulin M, Serum levels of immunoglobulin G and the CD4/CD8 ratio of study participants at baseline and at the end of the study in the placebo and BCO-5 treated groups. Values are expressed as mean ± SD. Differences were considered statistically significant at *p* < 0.05.

### 3.9. Influence on CD4+, CD8+ and CD4/CD8 ratio

Intra-group comparison of CD4+ and CD4/CD8 ratio exhibited a significant increase (*p* = 0.015 and *p* < 0.001respectively) in BCO-5 treated participants, whereas the placebo-supplemented group showed no significant effect (*p* > 0.05). CD8+ cells, on the other hand, showed a significant decrease (*p* = 0.043) compared to that at baseline.

Inter-group comparison revealed a significant increase in the CD4+ and CD4/CD8 ratio at the end of the study in BCO-5 treated participants. The effect size observed were: CD4+: 0.29 (95% CI: 765.07–819.02; *p* < 0.001) and CD4+/CD8+: 0.52 (95% CI: 1.85–2.08; *p* = 0.032) respectively. At the same time, CD8+ exhibited a much more significant decrease at the end of the study compared to the placebo, with a moderate effect size of 0.46 (95% CI: 387.90–425.91; *p* = 0.042) (Table 3).

#### 3.9.1. Influence on differential count

At the end of the study, supplementation with BCO-5 increased the number of leukocytes, lymphocytes, and monocytes in both intra- and inter-group analyses. The relative effect size observed were, respectively, 0.34 (95% CI: 6342.14–6921.85; *p* = 0.050), 0.57 (95% CI: 35.80–40.26; *p* = 0.009), and 0.10 (95% CI: 5.57–6.97; *p* < 0.001). Neutrophils and eosinophils, on the other hand, showed a significant decrease, with an effect size of 0.57 (95% CI: 51.51–55.45; *p* < 0.001) and 0.31 (95% CI: 1.61–2.33; *p* < 0.001) respectively. The basophil count showed no significant difference (0.001; 95% CI: 0.17–0.27; *p* = 0.294) (Table 4).

**Table 4:** Changes in differential count in participants from baseline to end of study in placebo and BCO-5 treated group and their respective effect size.

Parameters	Group	Baseline	End of study	Effect size
TLC (cells/cmm)	Placebo	6,072 ± 777.56	5,792 ± 776.17	0.34
	BCO-5	5,992 ± 707	6632 ± 702.21	
Neutrophils (%)	Placebo	66.24 ± 3.44	63.32 ± 3.59	0.57
	BCO-5	64.29 ± 3.99	53.48 ± 4.77	
Lymphocytes (%)	Placebo	28.68 ± 3.90	31.29 ± 3.66	0.57
	BCO-5	27.30 ± 4.83	38.04 ± 5.40	
Eosinophils (%)	Placebo	1.38 ± 0.71	1.44 ± 0.78	0.31
	BCO-5	2.75 ± 1.00	1.98 ± 0.87	
Monocyte (%)	Placebo	3.44 ± 1.83	3.68 ± 1.58	0.10
	BCO-5	5.42 ± 1.65	6.28 ± 1.70	
Basophils (%)	Placebo	0.26 ± 0.11	0.26 ± 0.09	0.001
	BCO-5	0.23 ± 0.12	0.23 ± 0.12	

A significance level *p* < 0.05 is considered as statistically different.

#### 3.9.2. Influence on cytokines- IL-1β, IL-2, and IL-10

Intra-group comparison of IL-1β, IL-2, and IL-10 showed no significant effect in the placebo. However, BCO-5 showed a significant increase in IL-1β (*p* < 0.001) and IL-2 (*p* < 0.001) and a significant decrease in IL-10 (*p* < 0.001) compared to their respective baseline values (Table 5).

**Table 5:** Changes in cytokines in participants from baseline to end of study in placebo and BCO-5 treated group and the *p*-values observed upon inter-group and intra-group comparison.

Parameters	Group	Baseline	End of study	*P*-values	
				Inter	Intra
IL-1β (pg/dL)	Placebo	2.41 ± 1.26	2.33 ± 1.79	<0.001	<0.001
	BCO-5	2.53 ± 1.20	6.75 ± 1.03		
IL-2 (pg/dL)	Placebo	1.91 ± 0.48	1.99 ± 0.75	<0.001	<0.001
	BCO-5	1.95 ± 0.57	5.21 ± 1.25		
IL-10 (pg/dL)	Placebo	25.59 ± 2.40	26.52 ± 4.44	<0.001	<0.001
	BCO-5	26.22 ± 3.40	13.76 ± 1.62		

A significance level *p* < 0.05 is considered as statistically different.

Inter-group comparisons at the end of the study revealed a significant increase in the levels of IL-1β (*p* < 0.001) and IL-2 (*p* < 0.001). The effect sizes of 0.75 (95% CI: 6.31–7.18) and 0.85 (95% CI: 4.69–5.73) were observed in IL-1β and IL-2, respectively. The decrease in IL-10 was also found to be significant, with an effect size of 0.88 (95% CI: 13.08–14.42; *p* < 0.001) (Table 5).

### 3.10. Safety and adverse events

The results of the hematological and biochemical analyses are given in Supplementary Table S1. All clinical laboratory parameters were within the normal range before and after the study, and there was no significant difference between the groups or within the groups (*p* > 0.05). There were six dropouts from the placebo group and four dropouts from the BCO-5 group. However, none of the dropouts were due to any adverse side effects due to personal reasons. Confirmation of acceptability/tolerability and safety was provided by the satisfaction ratings at the end of the study. It was found that 2% of the participants in the intervention group (17% in the placebo group) were discontented with capsule intake.

## 4. Discussion

The present study investigated the ability of a novel formulation of black cumin oil extract (BCO-5) to modulate stress, sleep, and immunity when supplemented at a dose of 200 mg/day for 90 days. The interconnection between stress, sleep, and immunity are well established (19). Its mechanisms of action and pathogenesis are also known (20). The novelty of the present study lies in the fact that this is the first report on the positive influence of a botanical extract, especially from a food component, on the safe modulation and alleviation of the stress-sleep-immunity axis. The rationale for the use of BCO-5 in the present study is a previous clinical study that employed polysomnography in healthy subjects with stress and sleep issues (12). Moreover, BCO-5 has also been shown to exhibit enhanced anti-inflammatory, anti-arthritic, acetylcholine esterase inhibitory, and neuroprotective effects in various preclinical studies (9, 10).

The study was conducted on healthy volunteers following a double-blind, placebo-controlled design. The total number of subjects (*n* = 72) enrolled and who completed the study was found to be statistically significant at the 80% power and 5% significance level. The baseline clinical laboratory tests and self-reported stress/sleep scores of the participants indicated that they were healthy but experienced significant sleep issues such as a non-restorative sleep (NRS) pattern due to stress/anxiety. The NRS is characterized by sleep disturbances and unsatisfactory sleep when awakening (21). Globally, more than 40% of the general population suffers from this sleep condition (22).

The primary objective of this study was to monitor the effect of BCO-5 on stress and sleep. To monitor stress, we used the well-validated short form of the Perceived Stress Scale (PSS-14), in which a higher score indicates a higher perception of stress (23). It provides a measure of the degree to which one’s life is affected by stress and anxiety. The baseline scores revealed that the participants experienced significant stress and sleep issues due to various reasons on their personal and professional fronts. The stress level (higher score) in the placebo and treated groups was not significantly different at baseline. Supplementation with BCO-5 significantly reduced (*p* < 0.001) the PSS scores on days 45 and 90 indicating its primary efficacy. Moreover, the decrease on day 90 was significantly higher than that on day 45 (*p* < 0.001). At the end of the study, the participants felt more relaxed and placid compared to the placebo group.

The PSQI, one of the most commonly used and well-validated self-reported rating scale, was employed to monitor the seven-component scores, that is sleep quality, sleep latency, sleep duration, sleep disturbance, sleep efficiency, medication use and daytime dysfunction as a measure of the overall sleep quality (15). We observed a significant increase with an effect size of 0.31 (*p* < 0.024) and 0.57 (*p* < 0.002) in sleep quality on days 45 and 90 which indicates peaceful and restorative sleep. Better sleep quality has been shown to improve health, reduce daytime sleepiness, and improve well-being, and psychological functioning (24). Sleep latency is defined as the time that a person falls asleep after turning off lights. Inter-group comparison on the 45th and 90th days revealed a significant decrease in latency scores, with an effect size of 0.31 (*p* = 0.03) and 0.82 (*p* < 0.001), indicating the positive influence of BCO-5. Sleep disturbance is a major issue affecting a high percentage of the population and is a disorder/difficulty in initiating and maintaining sleep. The observation that sleeps disturbance scores were significantly reduced upon supplementation with BCO-5, with an effect size of 0.19 (*p* = 0.030) and 0.20 (*p* = 0.043), respectively on days 45 and 90, further shows its positive impact on sleep. BCO-5 also improved sleep duration, as evidenced by the results with an overall effect size of 0.24 (*p* = 0.02) and 0.33 (*p* = 0.001) respectively on days 45 and 90. Sleep duration refers to sleep obtained, either nocturnal or within 24-h time (25), and it was correlated with an increased risk of developing hypertension, diabetes, obesity, depression, heart attack, and stroke (26).

Sleep efficiency, which is the ratio of total sleep time to the time spent in bed, is another important component of the PSQI that captures the core problem for those suffering from insomnia. Poor sleep efficiency can increase daytime sleepiness and contribute to sleep debt (27). Supplementation with BCO-5 significantly improved sleep efficacy with an effect size of 0.56 and 0.47 on days 45 and 90, respectively. Moreover, BCO-5 has also shown a significant decrease in daytime dysfunction and inability to perform daily functions. The correlation between PSQI and PSS-14 was further evaluated using Pearson’s correlation test and was found to be significant indicating the potential role of BCO-5 in reducing stress and improving sleep quality (−0.314; *p* < 0.05). In contrast, the placebo group showed a positive correlation at the end of the study (0.178; *p* = 0.357). Our findings were also in agreement with a previous report by Shelar et al. (28), who found a negative correlation between PSQI and PSS-14, reinforcing the relationship between reduced stress and increased sleep.

**Figure 7:**
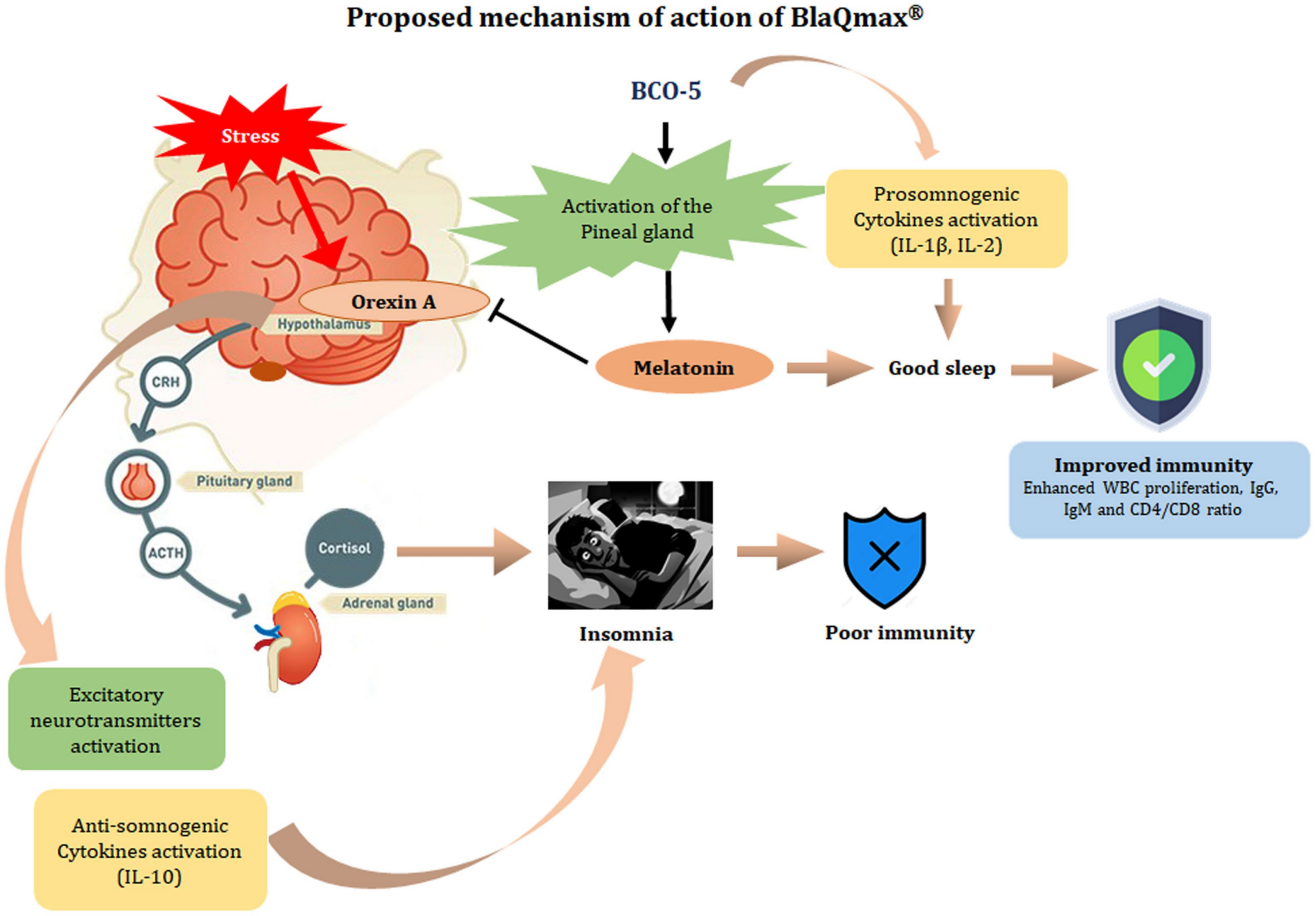
Proposed mechanism of action of BCO-5. Orexins (Orexin A and Orexin B) are the key neuropeptides produced in the hypothalamus and interact with the hypothalamic–pituitary–adrenal axis (HPA) to regulate sleep–wake cycle and neuroendocrine activation associated with stress. Orexins cause the release of corticotropin-releasing hormone (CRH) in the hypothalamus, which triggers the anterior pituitary to secrete adrenocortical-tropic hormone (ACTH), stimulating the release of cortisol – the major stress hormone, and promote wakefulness. In the nervous system, orexins result in the release of excitatory neurotransmitters such as dopamine, epinephrine, glutamate, and acetylcholine which further result in alertness/wakefulness. BCO-5 acts on the pineal gland and releases melatonin which acts as a natural inhibitor of orexins and thereby regulate HPA axis to reduce stress and promote sleep. In other words, BCO-5 act as a dual orexin receptor antagonist (DORA).

Furthermore, the measurement of salivary melatonin, cortisol, plasma orexin A, and cytokines provided an insight into the molecular basis of the action of BCO-5 on stress and sleep. Recently, there has been great interest in the development of Dual Orexin Receptor Antagonists (DORA) for treating chronic insomnia because orexin agonism during the day promotes wakefulness, and orexin receptor antagonists can promote sleep signals by enhancing melatonin levels (29, 30). Stress stimuli can generate orexins (orexin A and B) which express the corticotropin-releasing hormone (CRH). CRH binds to the anterior pituitary gland to release adrenocorticotropic hormone (ACTH), which further binds to its receptor in the adrenal cortex and releases cortisol, the major hormone produced in response to stress (31). Therefore, orexins act as molecular switches for the release of cortisol in response to a stress stimulus and regulate the sleep/wake cycle (Figure 7) (4). Melatonin, a hormone referred to as “the light of night,” has been established as a natural inhibitor of orexins; hence, it can reduce stress by reducing cortisol levels and improve sleep quality (32). In agreement with this molecular mechanism, our results showed a significant increase in the salivary melatonin levels among the BCO-5 group, with a large effect size of 0.85 (*p* = 0.009) and a decrease in cortisol level with a large effect size of 0.72 (*p* = 0.013). The ability of BCO-5 to increase melatonin levels may be explained on the basis of previous studies showing that black cumin increases the concentration of 5-hydroxy tryptophan and tryptophan in the rat brain and plasma (33). Tryptophan and 5-hydroxy tryptophan can increase the concentration of serotonin, the biosynthetic precursor of melatonin (34, 35). We also observed a significant reduction in the plasma concentration of orexin A among the BCO-5 participants (0.92; *p* < 0.001), indicating that BCO-5 acts as a natural DORA. The molecular mechanism of BCO-5 in stress and sleep are depicted in Figure 7.

Sleep loss has been reported to trigger stress and elevate the plasma levels of various cytokines (36). Cytokines that alter sleep in humans and animals have been identified as IL-1β, IL-2, IL-4, IL-10, IL-13, IL-15, TNF-α, IFN-γ, and macrophage inhibitory protein-1β (MIP-1β), in which IL-1β, IFN-γ, IL-2, and TNF-α are considered pro-somnogenic cytokines (supporting sleep) and IL-4, IL-15, IL-10, and IL-13 were observed with sleep deprivation (anti-somnogenic) (5); IL-1β, IL-2, and IL-10 are the mainly studied cytokines that are known to be involved in sleep regulation (37). Our findings showed an elevated level of pro-somnogenic cytokines IL-1β and IL-2 and a decrease in anti-somnogenic IL-10, indicating the positive effect of BCO-5 on sleep modulation. Previous studies have also reported elevated levels of IL-2 and IL-1β upon improved sleep and an elevation in IL-10 levels upon sleep disorders (19, 38, 39).

Sleep and immunity are also interconnected. Epidemiological studies have shown that poor sleep increases susceptibility to diseases (1, 5, 40). Various biochemical studies have demonstrated that sleep loss can alter immune responses in both animals and humans (41–43). Leukocyte proliferation, humoral immunity, cell-mediated immunity, and immunoglobulin levels are reduced in participants with prolonged sleep disorders (44). Two or three nights of sleep deprivation can significantly decrease the neutrophil, lymphocyte and leukocyte counts (40). Recently, it has also been shown that every 60-min increase in sleep duration was associated with a significant increase in total leukocytes, lymphocytes, neutrophils, and monocyte count (*p* < 0.001) (45). Our results for the differential count at baseline agree with this observation. We found that BCO-5 supplementation significantly enhanced leukocyte proliferation, as evident from the increase in the total leukocyte, lymphocytes, and monocytes counts.

Immunoglobulins (Ig) or antibodies are glycoproteins produced by plasma B cells which are critical for immune response and hence important markers of immunomodulatory effects. IgA, IgG, IgM, IgE, and IgD are immunoglobulins found in humans. IgG accounts for the most abundant immunoglobulin (75%) (46), whereas IgM is the largest antibody, accounting for approximately 5% of all antibodies in the serum. Immunoglobulins quickly recognize and initiate an immune response by directly neutralizing pathogens or clearing novel antigens (47, 48). Although various studies have attempted to correlate immunoglobulin levels with sleep disorders, the results are controversial and require further clarity. Everson reported a significant increase in IgG, IgM, and IgA levels after 3–5 days of sleep deprivation (49). However, other studies were unable to replicate this result and reported either no change or reduction in immunoglobulin levels upon sleep deprivation (40, 50). Our results indicated a significant increase in IgG and IgM levels in BCO-5 participants; however, the increase was within the normal range (48).

Cell-mediated immunity plays a critical role in antiviral responses (51). The absolute count of CD4+ T lymphocytes was significantly increased. This correlates with the increased levels of IL-2, a pro-somnogenic cytokine released by CD4+ cells. CD4+ cells are also known to promote immunoglobulins. However, a significant reduction was observed in the number of CD8 cells. The CD4/CD8 ratio is a direct marker of immune status, showing a decreasing trend in the placebo group and a significant increase in the BCO-5 group. In a previous study, Salem et al. reported a significant immunomodulatory effect of black cumin seed powder, with a significant increase in immunoglobulin, CD4+ cell absolute count, and lymphocyte counts (52). Certain animal studies have also reported immunomodulatory effects of black cumin (53–55).

The absence of significant side effects, adverse events, or toxic deviations in clinical laboratory parameters (biochemical/hematological) indicated its safety and suitability for supplementation. A detailed safety assessment of BCO-5 has recently been published, which also revealed no significant toxic effects (13). Acute and sub-chronic toxicity studies have also established the safety of BCO-5 and its optimized, safe dosage for human consumption of 900 mg/kg/day (56). The fact that more than 70% of the participants reported significant improvement in their sleep pattern by day 7 indicates the relatively fast action of BCO-5 as a natural stress/sleep aid.

The lack of an objective assessment of sleep parameters and the measurement of neurotransmitters and markers of innate immunity may be considered as limitation of the present study. Similarly, investigations of the bioavailability of the key bioactive molecules in BCO-5 would provide better insights into its mechanism of action.

## 5. Conclusion

In summary, the present randomized double-blinded placebo-controlled trial demonstrated the efficacy of a proprietary black cumin extract (BCO-5) to safely modulate the stress-sleep-immunity axis and further alleviate stress, establish restorative and restful sleep, and improve immunity among healthy subjects characterized by non-restorative sleep conditions. While PSS-14 demonstrated a significant reduction in stress, PSQI analysis established a positive effect of BCO-5 on sleep quality, sleep latency, sleep duration, and overall sleep efficiency when supplemented at a single dose of 200 mg/day. The percentage of participants who were satisfied with a single dose confirmed the rapid action of BCO-5. The improvements in stress and sleep reported by participants in the PSS-14 and PSQI questionnaires were in agreement with the changes in salivary melatonin and cortisol levels, plasma orexin, and cytokine levels. The influence of BCO-5 on immunity status was also clear from the differential counts, immunoglobulins, cytokines, and CD4+, CD8+, and CD4/CD8 ratios. The therapeutic potential of BCO-5 may be attributed to the pleiotropic mechanism of action that regulates the HPA axis and thereby modulates stress, sleep, and immunity functions, the three interconnected factors of optimized health, but with different pathways. Future studies should investigate the short-term effects of BCO-5 on various sleep problems by employing techniques such as actigraphy and its influence on anxiety, depression, and mood. Detailed investigation of the molecular mechanism of action is also recommended.

## Data availability statement

The original contributions presented in the study are included in the article/Supplementary material, further inquiries can be directed to the corresponding author.

## Ethics statement

The studies involving human participants were reviewed and approved by the Balagangadharanatha Swami Global Institute of Medical Sciences Institutional Ethics Committee. The patients/participants provided their written informed consent to participate in this study.

## Author contributions

MEM: recruitment and investigation of the trial. JT: conduct of the trial, data collection, and biochemical analysis. PP: drafting the original article. SD and PP: data analysis. MCM: review and editing of the original article. BP: principal investigator who supervised the study and was involved in the protocol development, data interpretation, review, and editing of the original article. All authors contributed to the article and approved the submitted version.

## Funding

The present study received funding from Akay Natural Ingredients, Cochin, India.

## Acknowledgments

The authors would like to express their sincere gratitude to the BGS Global Institute of Medical Sciences and CUSATECH Foundation, Cochin University of Science and Technology, Cochin, India, for their support and advice during the course of this study.

## Conflict of interest

BCO-5 used in the present study is a patented black cumin extract developed by Akay Natural Ingredients, Cochin, India and registered as BlaQmax^®^. PP and SD were employed by Akay Natural Ingredients. JT was employed by Leads Clinical Research and Bio Services Private Limited.

The remaining authors declare that the research was conducted in the absence of any commercial or financial relationships that could be construed as a potential conflict of interest.

## Publisher’s note

All claims expressed in this article are solely those of the authors and do not necessarily represent those of their affiliated organizations, or those of the publisher, the editors and the reviewers. Any product that may be evaluated in this article, or claim that may be made by its manufacturer, is not guaranteed or endorsed by the publisher.

## Footnotes

^1^ www.randomization.com
