# Supplementary material for: A proprietary black cumin oil extract (Nigella sativa) (BlaQmax®) modulates stress-sleep-immunity axis safely: Randomized double-blind placebo-controlled study
Source: Front Nutr. 2023 Apr 17;10:1152680. doi: 10.3389/fnut.2023.1152680 (PMC10149792; doi:10.3389/fnut.2023.1152680)
Supplement: Supplementary file 1 [file Data_Sheet_1.docx]

Supplementary Material

**A proprietary formulation of black cumin oil extract (*Nigella sativa*) (BlaQmax) modulates stress-sleep-immunity axis safely: Randomised double-blind placebo-controlled study**

**Mohan ME^1^, Jestin V. Thomas^2^, Mohind C Mohan^3^ Syam Das S^4^, Prathibha Prabhakaran^4^, Baby Chakrapani PS ^3,5^***

*** Correspondence:** Baby Chakrapani PS: chakrapani@cusat.ac.in, bcps80@gmail.com

**Table S1.** Changes in vital signs, demographic, anthropometric and safety parameters from baseline to end of study of in placebo and BCO-5 treated group

| **Parameters** | **Groups** | **Baseline** | **End of study** |
| --- | --- | --- | --- |
| Age (yr.) | Placebo | 35.83 ± 6.77 | |
|  | BCO-5 | 37.03 ± 6.11 | |
| BMI (Kg/m^2^) | Placebo | 24.12 ± 4.98 | 24.04 ± 4.90 |
|  | BCO-5 | 24.03 ± 4.31 | 24.00 ± 4.29 |
| Systolic BP (mmHg) | Placebo | 121.79 ± 5.45 | 123.17 ± 4.50 |
|  | BCO-5 | 122.16 ± 4.16 | 121.32 ± 3.42 |
| Diastolic BP (mmHg) | Placebo | 83.10 ± 6.24 | 82.58 ± 5.37 |
|  | BCO-5 | 83.35 ± 3.97 | 82.51 ± 3.15 |
| Pulse rate | Placebo | 78.85 ± 2.99 | 79.26 ± 2.75 |
|  | BCO-5 | 78.60 ± 4.07 | 78.73 ± 3.94 |
| Hb (g/dL) | Placebo | 13.30 ± 0.29 | 13.28 ± 0.43 |
|  | BCO-5 | 13.55 ± 0.60 | 13.37 ± 0.35 |
| RBC count (million/μL) | Placebo | 5.07 ± 0.42 | 5.06 ± 0.39 |
|  | BCO-5 | 5.16 ± 0.45 | 5.12 ± 0.43 |
| MCV (fL/red cell) | Placebo | 82.69 ± 3.39 | 83.70 ± 4.81 |
|  | BCO-5 | 83.80 ± 6.13 | 82.41 ± 2.90 |
| MCH (pg/cell) | Placebo | 27.15 ± 1.17 | 27.68 ± 1.06 |
|  | BCO-5 | 26.98 ± 1.75 | 27.15 ± 0.96 |
| ALT (U/L) | Placebo | 28.97 ± 8.82 | 28.13 ± 5.60 |
|  | BCO-5 | 30.04 ± 6.64 | 30.26 ± 7.70 |
| AST (U/L) | Placebo | 24.43 ± 5.14 | 24.24 ± 5.22 |
|  | BCO-5 | 24.79 ± 3.58 | 24.72 ± 3.47 |
| ALP (U/L) | Placebo | 84.90 ± 13.00 | 86.54 ± 13.41 |
|  | BCO-5 | 88.04 ± 15.90 | 86.82 ± 13.65 |
| Creatinine (mg/dL) | Placebo | 0.98 ± 0.11 | 0.96 ± 0.09 |
|  | BCO-5 | 0.93 ± 0.09 | 0.94 ± 0.09 |

BMI-body mass index, BP- blood pressure. Values are expressed as Mean ± SD. A significance level *P* < 0.05 is considered as statistically different and mentioned with a ‘*’.

**Table S2: Percentage difference and *P*- values of PSQI parameters upon inter and intragroup comparison**

| PSQI Parameter | Time | Percentage of  difference | *P*-values | |
| --- | --- | --- | --- | --- |
|  |  |  | Inter | Intra |
| Sleep quality | 45^th^ day | 30.96 | < 0.001 | < 0.001 |
|  | 90^th^ day | 48.94 | < 0.001 | < 0.001 |
| Sleep latency | 45^th^ day | 19.00 | 0.001 | < 0.001 |
|  | 90^th^ day | 58.01 | < 0.001 | < 0.001 |
| Sleep duration | 45^th^ day | 26.38 | < 0.001 | < 0.001 |
|  | 90^th^ day | 22.58 | < 0.001 | < 0.001 |
| Sleep efficiency | 45^th^ day | 65.46 | < 0.001 | < 0.001 |
|  | 90^th^ day | 76.50 | < 0.001 | < 0.001 |
| Sleep disturbance | 45^th^ day | 25.92 | < 0.001 | 0.002 |
|  | 90^th^ day | 29.26 | < 0.001 | < 0.001 |
| Daytime dysfunction | 45^th^ day | 33.90 | < 0.001 | < 0.001 |
|  | 90^th^ day | 51.39 | < 0.001 | < 0.001 |
| Total PSQI | 45^th^ day | 32.99 | < 0.001 | < 0.001 |
|  | 90^th^ day | 48.41 | < 0.001 | < 0.001 |

The values are expressed as difference in percentage between placebo and BCO-5 groups. A *P* < 0.05 is considered as statistically significant

**Table S3: Changes in PSS values and *P* values upon intra and intergroup comparison at baseline and end of study in placebo and BCO-5 treated groups**

| **Parameters** | **Group** | **Baseline** | **End of Study** | ***P*-values** | |
| --- | --- | --- | --- | --- | --- |
|  |  |  |  | **Intra** | **Inter** |
| PSS (45) | Placebo | 22.19 ± 3.35 | 20.32 ± 6.93 | 0.233 | 0.010 |
|  | BCO-5 | 22.39 ± 2.96 | 16.76 ± 3.18 | < 0.001 |  |
| PSS (90) | Placebo | 22.17 ± 3.40 | 18.62 ± 7.49 | 0.069 | < 0.001 |
|  | BCO-5 | 22.32 ± 3.02 | 10.39 ± 1.31 | < 0.001 |  |

Values are expressed as Mean ± SD. A *P* < 0.05 is considered as statistically significant
